# Adaptive Appraisals of Anxiety Moderate the Association between Cortisol Reactivity and Performance in Salary Negotiations

**DOI:** 10.1371/journal.pone.0167977

**Published:** 2016-12-16

**Authors:** Modupe Akinola, Ilona Fridman, Shira Mor, Michael W. Morris, Alia J. Crum

**Affiliations:** 1 Columbia Business School, Columbia University, New York, NY, United States of America; 2 Tel Aviv University, Tel Aviv, Israel; 3 Department of Psychology, Stanford University, Stanford, CA, United States of America; IRCCS Istituto Auxologico Italiano, ITALY

## Abstract

Prior research suggests that stress can be harmful in high-stakes contexts such as negotiations. However, few studies actually measure stress physiologically during negotiations, nor do studies offer interventions to combat the potential negative effects of heightened physiological responses in negotiation contexts. In the current research, we offer evidence that the negative effects of cortisol increases on negotiation performance can be reduced through a reappraisal of anxiety manipulation. We experimentally induced adaptive appraisals by randomly assigning 97 male and female participants to receive either instructions to appraise their anxiety as beneficial to the negotiation or no specific instructions on how to appraise the situation. We also measured participants’ cortisol responses prior to and following the negotiation. Results revealed that cortisol increases were positively related to negotiation performance for participants who were told to view anxiety as beneficial, and not detrimental, for negotiation performance (appraisal condition). In contrast, cortisol increases were negatively related to negotiation performance for participants given no instructions on appraising their anxiety (control condition). These findings offer a means through which to combat the potentially deleterious effects of heightened cortisol reactivity on negotiation outcomes.

## Introduction

Stress is often synonymous with poor performance in high-stakes contexts, such as negotiations. However, extant research begs a more nuanced understanding of the role of stress in negotiation performance. Although recent studies have found that inducing anxiety, which is often associated with stress [1], has negative consequences for negotiators [2, 3], one limitation of these studies is that they rely on self-reports, leaving open the question of how the actual biological experience of stress during a negotiation can influence negotiation outcomes. Further, research that examines the interplay between biological responses and psychological construals of emotions on negotiation performance is needed, consistent with evidence demonstrating that how one construes emotions and one’s bodily responses can influence behavior and performance [4–6]. In the current study, we measure cortisol responses during a negotiation to examine how neurobiological responses can differentially influence negotiation outcomes, depending on how individuals appraise the anxiety that can accompany stressful situations.

Cortisol, a catabolic hormone secreted by the hypothalamic pituitary adrenal (HPA) axis, is responsive to situations characterized by physical and psychological stress, uncertainty, social-evaluative threat, and uncontrollability, all of which can accompany negotiations [2, 7, 8]. Short-term increases in cortisol can be adaptive, preparing the body to respond to a stressor, yet prolonged cortisol elevation can have deleterious effects on the brain, behavior, and cognition [9]. Additionally, while some studies have associated cortisol increases with poorer performance, negative affect, withdrawal motivation, and higher demand relative to resource appraisals [10–15] others have shown positive associations between cortisol responses and performance. Cortisol increases can put the brain and body in an optimal condition to perform [16], recruit attentional resources [17], and boost memory and performance on cognitive tasks [18–19]. These finding are consistent with studies linking heightened sympathetic nervous system (SNS) responsiveness to improved cognitive performance in numerous domains [20–22].

These varied effects of cortisol reactivity on performance are often explained by how individuals construe the situation. Depending on personal coping strategies, early experiences, and the situational context, individuals could appraise the stressful situation they encounter either in an adaptive manner (e.g., an approach orientation where resources are perceived to meet demands), which can enhance positive affect, and boost motivation and cognitive performance, or in a maladaptive manner (e.g., an avoidance orientation where demands are perceived to exceed resources), resulting in reduced motivation and poorer cognitive performance [10, 23]. In addition, there are studies showing that appraisals of the experience of stress can influence performance. Emotion regulation research highlights that reappraising the experience of stress, by reinterpreting the situation one is facing, can promote adaptive responses to high arousal states [24, 25]. For example, participants instructed to think about their physiological responses to anxiety as beneficial to performance during a Graduate Records Examination (GRE) test exhibited elevated SNS responses and performed better on the GRE relative to control participants [4]. Other studies have replicated and extended this effect to show that interventions instructing participants to reappraise heightened physiological responses to stress as beneficial led to more approach-oriented behavior, more adaptive physiological responses, and improved performance, relative to instructions to reappraise arousal as harmful or control conditions with no interventions [24, 26, 27]. These findings demonstrate that physiological responses, coupled with adaptive appraisals, may be advantageous for performance.

The goal of the current research was to broaden our understanding of how the biological experience of negotiation may interact with psychological construals of anxiety during negotiations. In examining this topic, we advance research and theory on the effects of physiological responses, namely cortisol increases, on performance in three important ways. First, we challenge the common notion in negotiation literature that stress can hinder negotiation performance and offer an intervention that can potentially moderate the effect of cortisol increases on negotiation performance. Second, reappraisal theory to date has focused primarily on individual and not interpersonal performance contexts, which can evoke heightened anxiety and stress responses [7]. By shifting focus to interpersonal contexts, we extend appraisal theory in an effort to demonstrate the power of reappraisal in highly cognitively demanding interpersonal situations. Third, since negotiations are ubiquitous in daily life [28], and given extant research highlighting that women underperform relative to men in negotiations [29, 30] and report feeling more stress in negotiations than do men [31, 2], identifying interventions that can mitigate the performance lowering effects of stress in negotiations for both men and women is of practical importance.

In the current study, we manipulated whether participants received instructions to reappraise their anxiety as a tool to help them maximize performance in a salary negotiation (appraisal condition) or received no instructions about the effects of anxiety on negotiation performance (control condition). We also measured participants’ cortisol responses prior to and following the negotiation. We predicted that cortisol increases would be associated with higher negotiation outcomes for individuals instructed to appraise their anxiety as beneficial for performance, and with lower negotiation outcomes for those receiving no intervention. Below we report all measures, manipulations, and exclusions used in the study.

## Methods

### Participants

Participants were 110 male and female (59% female) university graduate and undergraduate students (Mean age: 20 years; *SD*: 4 years; 28% Caucasian, 10% African-American, 48% Asian, 12% Latino, and 2% other). Undergraduate (N = 48) and graduate (N = 49) students were equally distributed across condition based on random assignment (appraisal: 52% undergraduate; control: 47% graduate). A power analysis based on the average effect size found in previous appraisal manipulations let to a targeted sample of 50 participants per condition. The experiment was advertised as a training opportunity for future job negotiations and students were compensated $8 for their participation. Seven participants did not reach an agreement with the recruiter and were excluded from the analysis consistent with prior research [32]. In addition, for six participants, cortisol results were incomplete (e.g., the first or second saliva sample analysis was missing) due to logistic and assaying errors. The final analyses therefore included 97 participants (Mean age: 24 years; *SD*: 4 years; 31% Caucasian, 8% African-American, 47% Asian, 11% Latino, and 3% other). This research was approved by the Columbia University IRB (#IRB-AAAE9335). The rights of the participants were protected, and applicable guidelines for research with human subjects were followed.

### Procedure

Participants arrived at the laboratory during the afternoon hours of 12 to 6pm. Upon arrival, participants signed consent forms, answered questions regarding their health behaviors, and provided a baseline saliva sample by expectorating into a cryovial tube. Participants were told that they would engage in a salary negotiation for a job at a consulting firm and randomly assigned to either an appraisal condition, where they were told that feeling anxious can enhance negotiation focus, or a control condition, where they were told nothing about the benefits of anxiety for negotiation. Participants were then escorted into an office, introduced to a white male recruiter (confederate), and engaged in a salary negotiation. Following the negotiation, participants completed a post-negotiation survey then provided a second saliva sample timed approximately fifteen minutes after the negotiation ended, capturing stress responses at the height of the negotiation [33].

### Materials and Measures

#### Health Behaviors

Participants completed a health behaviors questionnaire assessing their caffeine and nicotine intake, sleep and wake time, exercise levels, and gum health. These measures served as biological covariates and were used as statistical controls in all analyses [33, 34].

#### Saliva Sampling and Hormone Assays

Saliva samples were obtained using polystyrene tubes prior to and following the negotiation. Upon completion of the study, saliva samples were stored in a -20°C freezer until shipped overnight on dry ice to a laboratory in College Park, PA (Salimetrics). Saliva samples were assayed for cortisol using a highly sensitive enzyme immunoassay (Salimetrics). Cortisol levels were in the normal ranges produced by Salimetrics. Average intra- and inter-assay coefficients of variation were 5.2% and 5.7%, respectively.

#### Appraisal Manipulation

Participants were randomly assigned to either a control condition or an appraisal condition prior to engaging in the negotiation. These conditions differed only in the instructions participants were given. The experimenter first told all participants the following:

“One goal of this research is to examine how physiological arousal during a negotiation correlates with negotiation performance. Because people vary in how anxious they are during negotiations, the saliva samples we are collecting will be analyzed for certain hormones that indicate your arousal level while taking today’s test. We will be examining the degree to which your arousal levels predict the outcome of the negotiation.”

In the appraisal condition, the experimenter followed this statement with:

“People often think that feeling anxious while negotiating will make them do poorly on the negotiation. However, recent research suggests that anxiety doesn’t hurt performance on negotiation and in some cases can help performance. In other words, people who feel anxious during a negotiation might actually do better. This means that you shouldn’t feel concerned if you do feel anxious while engaging in today’s negotiation. If you find yourself feeling anxious, you might simply remind yourself that your anxiety could be helping you do well.” (adapted from [4]).

In the control condition, the experimenter followed with:

“You will be entering the room with the recruiter shortly. Remember that you are a senior at [your] University majoring in economics currently in search of a job for after graduation. You have read through the preparation sheet and should have filled out the preparation questions.”

Following these instruction, in both conditions, the experimenter then entered the room and escorted the participant to the negotiating room.

#### Negotiation Exercise

To model a salary negotiation, participants received materials that included a resume of a senior undergraduate student, an open job description for a position in a hypothetical consulting firm, and a brief review of current job postings advertising a starting salary between $43,000 and $87,500 for consulting positions. Participants reviewed these materials alone and were informed that if they failed to reach an agreement with the recruiter, their backup option was a job at a less preferred company with a salary of $32,000. Participants were not given a time limit for the negotiation. Participants were then escorted into a room where a white male recruiter was seated. The recruiter was a confederate who was blind to experimental condition and to the study hypotheses. Consistent with prior negotiation studies [35, 36], the recruiter’s responses were predetermined and the same confederate was used for all participants. The confederate was a military veteran with ten years of corporate experience and was therefore older than the majority of participants, allowing him to realistically portray a corporate recruiter. After an introductory conversation, the negotiation proceeded in a series of rounds of offers. The recruiter always started with an offer of $45,000. If the participant counter-offered, the recruiter contemplated the offer then raised his offer by $2,000, providing a scripted response and rationale for the new salary amount. This process continued with salary increments of $2,000 until the participant either accepted an offer or they reached the maximum salary of $57,000 (six rounds of offers). The average negotiated salary was $49,730 and the average time spent negotiating was 12 minutes.

#### Post-Negotiation Survey

Following the negotiation, participants reported the agreed upon salary and subjectively assessed their negotiation experience. The agreed upon salary was corroborated by the confederate. Participants also responded to questions regarding their experience during the negotiation, including how nervous and stressed, confident, uncomfortable, relaxed, eager to negotiate, and assertive they felt. They were also asked if they desired to receive feedback. We do not report on these measures as subjective assessments are not the key focus of our research question. However, post-hoc analysis, specifically examining self-reported stress levels revealed no significant relationship between self-reported stress and negotiation outcome or cortisol response in either condition. These post-hoc findings are consistent with research suggesting that the motives captured by hormonal measures may not be easily detected by self-report measures [37] and highlight the relative independence of responses across non-biological and biological systems [38, 39].

## Results

We examined the effects of appraisal condition, cortisol increase, and their interaction on negotiation outcome using the bootstrapping procedure PROCESS, Model 1 [40] (See Table 1 for correlations and descriptive statistics). To measure cortisol increase, we used post-negotiation cortisol levels as an independent variable and baseline cortisol levels as a covariate [41]. Additionally, given research highlighting gender differences in negotiation [29, 30], to rule out any gender differences in our analysis, we first examined the gender x appraisal condition x cortisol level interaction on negotiation outcomes. We observed no gender effects and therefore used gender as a covariate in our analyses.

**Table 1:** Correlations and Descriptive Statistics by Condition.

Control Condition					
	1	2	M	SD	N
1. Cortisol (pre-negotiation)	-		0.14	0.08	46
2. Cortisol (post-negotiation)	.34*	-	0.16	0.10	46
3. Negotiation Outcome	.01	-.14	$49, 570.00	$3,124.00	46
Appraisal Condition					
	1	2	M	SD	N
1. Cortisol (pre-negotiation)	-		0.14	0.07	51
2. Cortisol (post-negotiation)	.60**	-	0.16	0.11	51
3. Negotiation Outcome	.03	.14	$49,860.00	$2,919.00	51

Note: Cortisol units are μg/dL.

*p < .05

**p < .01.

**Fig 1:**
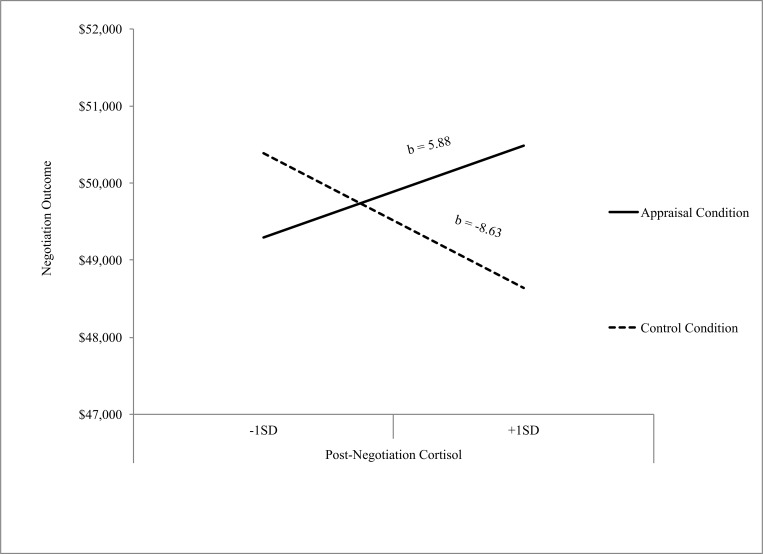
Relationship between Post-Negotiation Cortisol Levels and Negotiation Performance by Condition, Controlling for Pre-Negotiation Cortisol. Note: Cortisol units are μg/dL. Slopes are reported as unstandardized betas.

Next, we turned to our key research question: does reappraising anxiety in an adaptive manner moderate the effect of cortisol increases on negotiation performance? We observed no main effect of cortisol increase on negotiation outcome, *b* = -8.64, *r*^*2*^_change_ = .05, *p =* .11, 95% CI:[-19.23, 1.95], nor was there a main effect of appraisal condition on negotiation outcome, *b* = -1.92, *r*^*2*^_change_ = .05, *p =* .13, 95% CI:[-4.44, 0.59]. However, as predicted, the interaction between cortisol increase and appraisal condition was significant, *b* = 14.52, *r*^*2*^_change_ = .05, *p =* .04, 95% CI:[0.81, 28.22] (Fig 1). Participants with high post-negotiation cortisol levels and in the appraisal condition had higher negotiation outcomes (*M*_*appraisal*_ = $50,486), *b =* 1.85, *se =* 0.95, *t*(85) = 1.94, *p =* .06, 95% CI:[-0.05, 3.74] as compared to participants in the control condition (*M*_*control*_ = $48,640). This relationship was not observed for participants with low post-negotiation cortisol levels (*M*_*apprasial*_ = $49,295; *M*_*control*_ = $50,388), *b =* -1.09, *se =* 0.95, *t*(85) = -1.15, *p =* .25, 95% CI:[-2.97, 0.79].

## Discussion

The current research offers evidence that a simple intervention prompting participants to view their anxiety as beneficial can not only mitigate the negative effect of cortisol increases on performance outcomes but can actually reverse it: participants with high cortisol responses who appraised their anxiety as beneficial performed better in the negotiation and walked away with higher salaries. These implications are aligned with the growing body of research demonstrating that appraisals of stress play a pivotal role in determining whether the effects of stress will be enhancing or debilitating (e.g., [27, 4]). Importantly, our results suggest that a simple intervention intended to engender adaptive appraisals was sufficient to capture the enhancing effects of heightened physiological responses in the context of negotiation. Moreover, given extant research on the benefit of reappraisals during stressful contexts focus more on individual versus interpersonal performance contexts, our results offer an important theoretical perspective by suggesting that reappraisal manipulations also function effectively in interpersonal negotiation situations. From a practical perspective, our findings imply that negotiators need not be distracted if they feel anxious and experience cortisol increases during a negotiation. Rather, they should interpret this anxiety as having enhancing properties instead of being detrimental to the desired outcome. Future research should continue to explore how attributions of anxiety may positively influence outcomes in a broad range of motivated performance situations, as well as in a variety of interpersonal situations including high stakes negotiations. Additionally, future research should consider using research designs that measure cortisol levels more frequently to get a more fine-grained sense of reactivity throughout the negotiation, as well as measure self-reports of anxiety to offer greater insight into how reappraisals of anxiety directly influence anxiety levels.

It is important to note that we observed only a moderate cortisol increase during the negotiation which could have been driven by the type of negotiation used in our study design. We intentionally included a salary negotiation with a confederate to offer more experimental control, but the simulated nature of this design might have dampened cortisol responsiveness as the structured bidding format may have been less stressful than a free-form conversation often seen in real world negotiation contexts. Future research is needed to test the reliability of these findings in naturalistic settings, such as negotiating for a raise or a promotion, that are more likely to evoke a heightened cortisol response. One question to address specifically is whether the nature of the relationship between cortisol increases and negotiation outcomes is influenced by the magnitude of the cortisol response. Our results show a linear relationship between cortisol increases and negotiation outcomes. However, this relationship may take on a curvilinear form under more extreme stress.

Additionally, we chose a zero-sum negotiation where the parties had preferences that were in complete opposition to each other; the participant wants to extract the highest salary possible from the recruiter, while the recruiter wants to give away as little money as possible. In more dynamic negotiations with multiple issues and opportunities to give and take with joint gains, we might also expect to see heightened cortisol responses. Being highly collaborative in nature, multi-issue negotiations are cognitively taxing and require negotiators to consistently juggle multiple issues and interests, potentially heightening stress levels. Therefore future research should vary the type of negotiation and even the nature of the relationship between negotiators in an effort to examine the role adaptive appraisals of the negotiation might play in various negotiation contexts. Finally, while this study was not designed to test for gender differences in negotiation performance, the preponderance of research on this topic [31, 32, 42, 43] begs for future (adequately powered) studies that investigate whether reappraisal of anxiety interventions may indeed help to enhance women’s performance in negotiations relative to men.

## Conclusions

Taken together, the present findings suggest that cortisol increases may in fact have enhancing properties in negotiations when accompanied by an adaptive appraisal of the emotions that may accompany the negotiation experience. As such, future research using both correlational and experimental methods and employing biological measures is needed to further our understanding of how adaptive appraisals can enhance performance in high-stakes interpersonal contexts like negotiations.
